# Transitioning to the “new normal” of learning in unpredictable times: pedagogical practices and learning performance in *fully online* flipped classrooms

**DOI:** 10.1186/s41239-020-00234-x

**Published:** 2020-12-21

**Authors:** Khe Foon Hew, Chengyuan Jia, Donn Emmanuel Gonda, Shurui Bai

**Affiliations:** Faculty of Education, The University of Hong Kong, Pok Fu Lam, Hong Kong SAR

**Keywords:** Pedagogy, Online flipped classroom, Flipped learning, Good practices

## Abstract

The COVID-19 outbreak has compelled many universities to immediately switch to the online delivery of lessons. Many instructors, however, have found developing effective online lessons in a very short period of time very stressful and difficult. This study describes how we successfully addressed this crisis by transforming two conventional flipped classes into *fully online* flipped classes with the help of a cloud-based video conferencing app. As in a conventional flipped course, in a fully online flipped course students are encouraged to complete online pre-class work. But unlike in the conventional flipped approach, students do not subsequently meet face-to-face in physical classrooms, but rather online. This study examines the effect of fully online flipped classrooms on student learning performance in two stages. In Stage One, we explain how we drew on the 5E framework to design two conventional flipped classes. The 5E framework consists of five phases—Engage, Explore, Explain, Elaborate, and Evaluate. In Stage Two, we describe how we transformed the two conventional flipped classes into fully online flipped classes. Quantitative analyses of students’ final course marks reveal that the participants in the fully online flipped classes performed as effectively as participants in the conventional flipped learning classes. Our qualitative analyses of student and staff reflection data identify seven good practices for videoconferencing*-*assisted online flipped classrooms.

## Introduction

“It’s now painfully clear that schools ought to have had more robust disaster-preparedness plans in place in the event of interruptions in their campus operations. But because many schools did not have such plans in place…online learning is about to get a bad reputation at many campuses, I suspect.” Michael Horn, cited in Lederman (2020), ‘Inside Higher Ed’.

In early January 2020, scientists identified a new infectious disease caused by a novel coronavirus. Since then, the COVID-19 pandemic has caused widespread disruptions to schools and universities. According to UNESCO, as of April 10, 2020, more than 188 countries had implemented nationwide school and university closures, impacting over 91% of the world’s student population (UNESCO n.d.).

During these school closures, all face-to-face lessons were cancelled, compelling many institutions, including our own university, to immediately transition from face-to-face in-person learning to completely online lessons. The abrupt switch to fully online learning has been particularly stressful for many instructors and students who prefer in-person instruction. Online learning is often stigmatized as a weaker option that provides a lower quality education than in-person face-to-face learning (Hodges et al. 2020). Indeed, such negative attitudes to fully online learning were revealed by a large EDUCAUSE survey (Pomerantz and Brooks 2017). The survey of 11,141 faculty members from 131 U.S. institutions found that only 9% of faculty prefer to teach a fully online course. In other words, a whopping 91% of faculty do not wish to teach in a completely online environment. Students’ opinions of fully online courses are not much better; a recent student survey by EDUCAUSE of more than 40,000 students across 118 American universities revealed that as many as 70% of the respondents mostly or completely prefer face-to-face learning environments (Gierdowski 2019).

Clearly, many faculty members and students do not see the value of fully online learning, despite the fact that online learning has been around for many decades. During the current health crisis, many instructors have had to improvise quick online learning solutions (Hodges et al. 2020). For example, in our own university, there are anecdotal reports of a myriad of emergency online methods. Some instructors, for example, merely uploaded their PowerPoint slides or papers onto a learning management system such as Moodle and asked students to read them on their own. Any questions were asked asynchronously on the Moodle forum. Other instructors recorded their own lectures (usually at least one hour long) and asked students to asynchronously watch the video lectures and then ask individual questions later. Still others talked for more than two hours via synchronous video platforms watched by students in their own homes. Although these online methods may be an efficient method of delivering content, they are not particularly effective in promoting active learning and interest (Bates and Galloway 2012). As one student remarked, “Sitting in front of my computer to watch a 2-h live lecture without any active learning activities such as group work is pretty boring!” Indeed, without any active learning activities such as peer interaction, a fully online course will feel more like an interactive book than a classroom (Sutterlin 2018).

Well-planned active online learning lessons are markedly different from the emergency online teaching offered in response to a crisis (Hodges et al. 2020). One promising strategy for promoting online active learning is the *fully online flipped classroom* pedagogical approach, hereafter referred to as the online flipped classroom approach. An online flipped classroom is a variant of the conventional flipped model. A conventional flipped classroom model consists of online learning of basic concepts before class, followed by face-to-face learning activities (Bishop and Verleger 2013). The conventional flipped model has become very popular in recent years due to its association with active learning, which emphasizes students’ active learning (Xiu and Thompson 2020). Active learning activities such as peer discussions can help students construct better understandings of the subject material (Deslauriers et al. 2019). Recent meta-analyses have provided consistent overall support for the superiority of the conventional flipped classroom approach over traditional learning for enhancing student learning (e.g., Låg and Sæle 2019; Lo and Hew 2019; Shi et al. 2019; van Alten et al. 2019).

The online flipped classroom is similar to the conventional flipped classroom model in that students are encouraged to prepare for class by completing some pre-class activities (e.g., watching video lectures, completing quizzes). However, unlike the conventional flipped classroom approach, students in online flipped classrooms do not meet face-to-face, but online (Stohr et al. 2020). Although the online flipped classroom appears to be gathering momentum in higher education, very few studies have examined its effectiveness (for an exception, see Stohr et al. 2020, who compared the online flipped classroom format with a conventional non-flipped teaching format). So far, we are not cognizant of any research that evaluated the efficacy of the fully online flipped classroom relative to the conventional flipped classroom. Establishing the effectiveness of online flipped classrooms is important, as practitioners need to know whether this active learning approach can be used during prolonged school closures.

Against this backdrop, this study compares the effects of online flipped classrooms versus conventional flipped classrooms on student learning outcomes. To this end, two conventional flipped classes in the Faculty of Education are transformed into online flipped classrooms. Students in both the online and flipped classes participated in the online pre-class activity asynchronously using a learning management system. However, students in the online flipped classes joined the online in-class learning synchronously using a video conferencing app whereas their counterparts in the conventional flipped classes attended face-to-face classes. The online flipped courses were designed using the 5E conceptual framework and used a cloud-based video conferencing app. We used the Zoom application after careful consideration of many different videoconferencing platforms. Our reasons for doing so are given in the Section of “Stage Two: Transforming conventional flipped classes into online flipped classes”.

The 5E framework consists of five phases—Engage, Explore, Explain, Elaborate, and Evaluate (Bybee et al. 2006).

- Engage—The first phase aims to engage students in the learning process. Methods to engage students usually include using a real-world scenario, or problem, asking students questions that allow them to brainstorm or think critically, and helping them to create connections to their past experiences.
- Explore—In the exploration phase, the teacher, who works as a facilitator or coach, gives the students time and opportunity to explore the content and construct their own understanding of the topic at hand.
- Explain—This phase starts with students attempting to explain specific aspects of the engagement and exploration experiences. Based on these explanations, the teacher introduces terminology in a direct and explicit manner to facilitate concept building.
- Elaborate—In this phase, the teacher provided more detailed information about the subject content through the use of mini lectures and/or whole class discussions. Students are also given the opportunity to apply what they have learned and receive feedback from the teacher and their peers.
- Evaluate—Formative assessments (e.g., quizzes) can be used to evaluate students’ mastery of the subject material at the beginning and throughout the 5E phases, and teachers can complete a summative assessment after the elaboration phase (e.g., final exams).

We adopted the 5E framework for the following reasons. First, the 5E framework, which is based on various educational theories and models (e.g., Herbart’s instructional model, Dewey’s instructional model, Atkin-Karplus Learning Cycle) (Bybee et al. 2006), provides a sound instructional sequence for designing a course and planning activities. The 5E framework can help instructors organize and integrate both the in-class and out-of-class learning activities (Lo 2017).

Second, previous research has shown the positive effect of the 5E framework on student achievement. These positive effects were initially established in science education (e.g., Akar 2005; Boddy et al. 2003). Recently, the 5E model has yielded positive results when applied to various subject areas and when used to design inquiry- and interaction-based learning activities. Mullins (2017), for example, found that undergraduate students in a 5E-supported class outperformed their peers in a traditional lecture setting. Hew et al. (2018) designed two postgraduate courses based on the 5E model in order to foster students’ active learning. Ninety-two percent of the participants agreed that the 5E supported courses were more engaging than traditional classroom instruction.

The rest of this paper is structured as follows. First, we describe our study design and methodology. This is followed by a description of our two stages of research. In Stage One, we explain how we use the 5E framework to design our two conventional flipped classes; In Stage Two, we describe how we transformed the two conventional flipped classes into fully online flipped classes, using a cloud-based video conferencing app. We describe the various pedagogical practices that Zoom videoconferencing can facilitate before and during online flipped classes. In this paper, we use the term “pedagogical practices” to refer to specific activities that are used to structure teaching and learning. This study is guided by the following two questions.

Research question 1: What effect does the change from a conventional flipped classroom format to an online flipped format have on student learning performance?
Research question 2: What are the good practices for videoconferencing*-*assisted online flipped classrooms, as perceived by students and/or teaching staff?

## Method

This study was conducted in a large public Asian university. Four classes were involved: (a) conventional flipped Course 1, (b) conventional flipped Course 2, (c) online flipped Course 1, and (d) online flipped Course 2. Conventional flipped Courses 1 and 2 were the *control* group. Online flipped Courses 1 and 2 were the *experimental* group. To avoid any potential instructor confounding bias, the same professor and teaching assistants (TAs) taught the conventional and online flipped formats of each class. Ethical approval to conduct the study was obtained from the Institutional Review Board at the University of Hong Kong and consent forms from all participants in the study were collected.

### Data collection and analysis

To reiterate, this study had two purposes: (a) to determine the effect of an online flipped classroom on student learning performance as determined by student final course marks, and (b) to determine good practices for videoconferencing*-*assisted online flipped classrooms, as perceived by the participants (students and teaching staff). We adopted a mixed methods involving quantitative and qualitative approaches to provide a deeper understanding of the research problem (Ivankova et al. 2006).

**Fig. 1:**
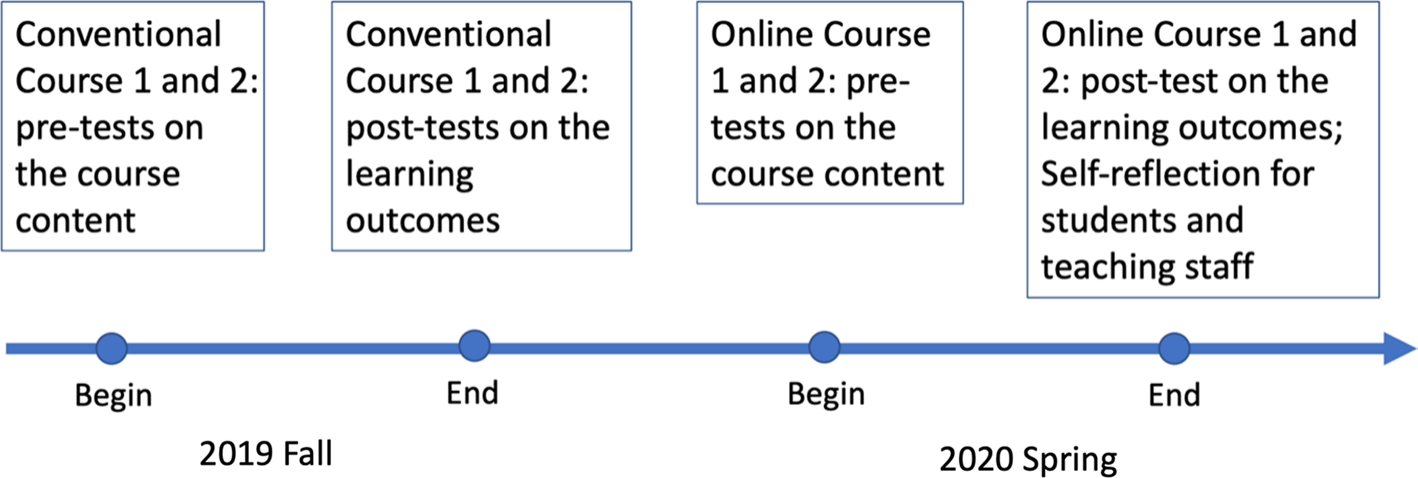
Timeline of data collection: 2019 Fall (before the pandemic), 2020 Spring (during the pandemic)

The data collection spanned across two semesters, which corresponded to the aforementioned two stages of the research. The conventional flipped classes were implemented in conventional flipped Courses 1 and 2 during the semester of 2019 Fall before the pandemic (Stage One). Due to the outbreak of Covid-19, all courses were required to be delivered online in our university in the 2020 Spring semester. Therefore, the online flipped classes were conducted in online flipped Courses 1 and 2 during the pandemic in 2020 Spring (Stage Two). Students’ knowledge and skills of the course content were checked at the beginning of the each course. Students final course marks in each course were collected and used as measure of the student learning outcomes at the end of the semester (See Fig. 1 for the research timeline).

To address the first purpose, we compared the students’ final course marks in the online flipped classrooms and conventional flipped classrooms. Quantitative data from 99 students were collected (see Table 1). We used the students’ final course marks to measure performance.

**Table 1:** Study samples

Format	Course	
	Course 1: E-learning strategies	Course 2: Engaging adult learners
Conventional flipped format	*N* = 23 (Female students = 20) Taught by Professor A and Teaching Assistant B	*N* = 25 (Female students = 20) Taught by Professor A and Teaching Assistant C
Online flipped format	*N* = 26 (Female students = 23) Taught by Professor A and Teaching Assistant B	*N* = 25 (Female students = 22) Taught by Professor A and Teaching Assistant C

To identify the perceived good practices for videoconferencing*-*assisted online flipped classrooms, we invited students and the teaching staff to complete a self-reflection exercise based on the following question: “What do you perceive as good practices in a videoconferencing-supported online flipped classroom?” The qualitative data collected from students and instructors were analyzed as follows. The first step was an initial reading of all of the response data to obtain an overall impression. The first author then applied the grounded approach (Strauss and Corbin 1990) to the qualitative data to generate relevant codes. Similar codes were organized into themes. In order to increase the consistency of coding, several exemplary quotes that clearly illustrated each constructed theme were identified. We also allowed new themes (if any) to emerge inductively during the coding process. The second author coded the data. There was perfect agreement with the coding. Table 2 summarizes how the data for each research question were collected and analyzed.

**Table 2:** Summary of research questions, data collection, and analyses

Research question	Data source	Data analysis
RQ 1: What effect does the change from a conventional flipped classroom format to an online flipped format have on student learning performance?	Student final course marks	Descriptive statistics, Inferential statistics
RQ 2: What are the good practices for videoconferencing*-*assisted online flipped classrooms, as perceived by students and teaching staff?	Student self-reflection, Instructor’s and teaching assistants’ self-reflection	Content analysis using the grounded approach

## Stage one: designing conventional flipped classes using the 5E framework

**Fig. 2:**
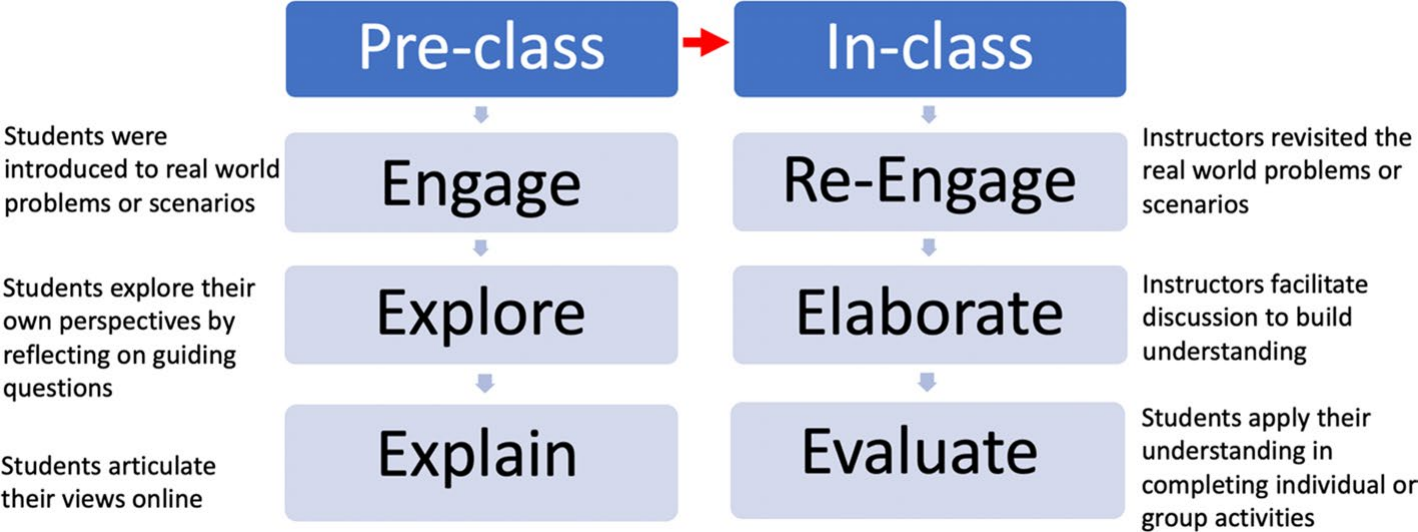
5E framework used to design the two conventional flipped classes

In this section, we first describe how we use the 5E framework to design our two conventional flipped classes (Course 1: *E-Learning Strategies*, and Course 2: *Engaging Adult Learners*). In the next section, we describe how we transform these two conventional flipped classes into fully online flipped classes. Figure 2 shows the 5E framework that guided our design of the conventional flipped classes. Table 3 shows some of the teaching and learning activities used in each of the 5E phases.

**Table 3:** Phases of the 5E framework and examples of activities in a conventional flipped classroom

5E phase	Component	Teaching and learning activities (example)	Mode
Engage	Pre-class	Depict real-life situations (videos, case-studies); trigger learner interest in the subject matter	Online (asynchronous)
Explore	Pre-class	Learner completes self-reflection (forum) Learner asks questions if necessary (WeChat)	Online (asynchronous)
Explain	Pre-class	Learner articulates own opinions (forum) Learner participates in focused peer discussion on specific issues/topics (forum) Learner asks questions if necessary (WeChat)	Online (asynchronous)
Elaborate	In-class	Teacher-led class discussion on issues to build understanding; teacher demonstration of strategies (if necessary)	Face-to-face
Evaluate	In-class	Student individual/group work and presentation, followed by teacher and peer feedback	Face-to-face

### Conventional flipped course 1: E-learning strategies

**Fig. 3:**
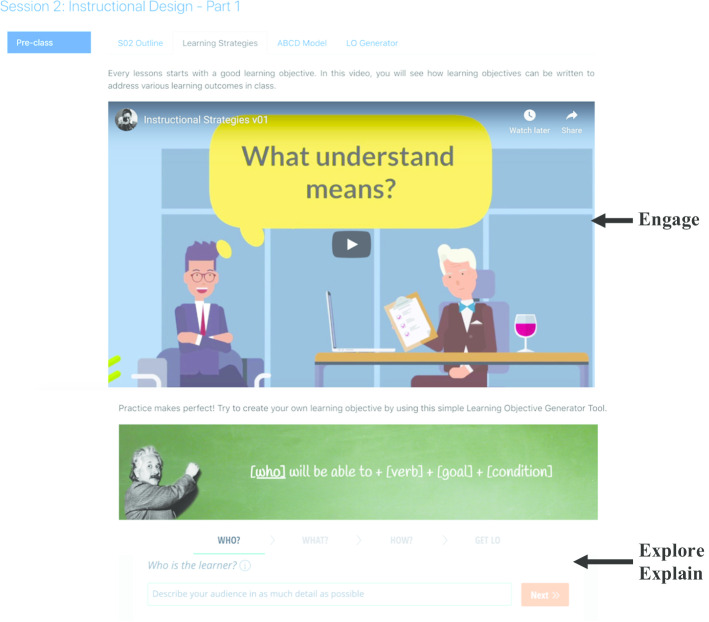
Example of a pre-class activity in Course 1

This course discussed the various e-learning strategies that can be employed to foster six types of learning, including problem-solving, attitude learning, factual learning, concept learning, procedural learning, and principle learning. There were eight sessions in the course. The first seven sessions were flipped—each consisting of an online pre-class learning component and a 3-h face-to-face in-class component. The last session was devoted to students’ presentations. Figure 3 shows an example of how the 5E framework was used in Course 1.

For instance, in the pre-class phase of *Session 2: Instructional Design—Part 1*, we posted a video that posed the question “What do we mean by ‘understand’”. This video **engaged** students’ curiosity about the importance of writing clear and measurable learning objectives. The instructor in the video highlighted the pitfalls of using vague words such as “know” and “understand” when writing learning objectives. Students then **explored** and **explained** their own individual learning objectives using the ABCD model (audience, behavior, condition, degree). Students were able to use a mobile instant messaging (MIM) app such as WeChat to ask questions of their peers or instructor. When a message arrived, a notification appeared on the receiver’s phone screen, encouraging timely feedback and frequent interaction (Rosenfeld et al. 2018).

**Fig. 4:**
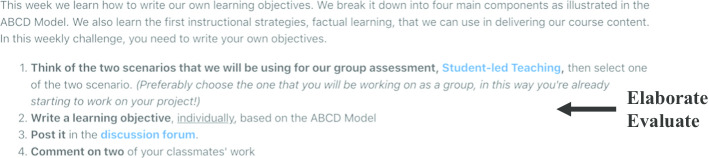
Example of an in-class activity in Course 1

During the face-to-face in-class session, the instructor re-engaged students’ attention by discussing basic instructional design issues such as “How do we write good lesson objectives?” The instructor conducted short debriefing sessions to discuss the strengths and weaknesses of students’ pre-class work. The instructor also facilitated class or small group discussions to build students’ understanding of how to write measurable lesson objectives that help students to achieve specific learning outcomes (e.g., factual learning). These discussions allowed students to **elaborate** on good lesson objectives practices. To **evaluate** the students’ understanding, the instructor asked them to work in groups of four on an instructional design scenario (e.g., teaching participants how to deal with angry customers), and then write a learning objective for the lesson in an online forum; their peers then commented on the posted learning objectives (Fig. 4).

### Conventional flipped course 2: engaging adult learners

**Fig. 5:**
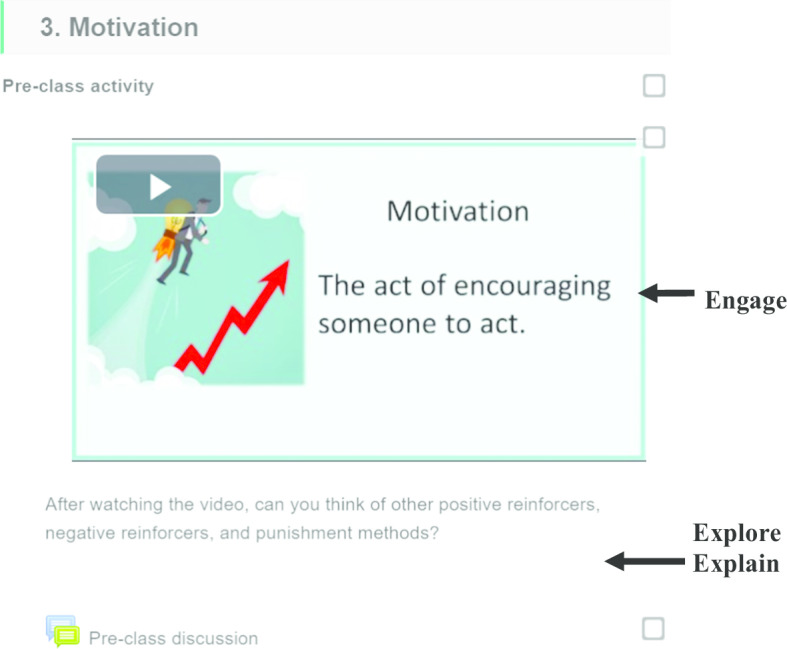
Example of a pre-class activity in Course 2

This course discussed the key principles of adult learning, as well as strategies used in adult education (e.g., transformational learning theory). There were eight sessions in the course, each session lasted three hours. An example of how the 5E instructional model was used is shown in Fig. 5.

For example, in the pre-class session for *Session 3: Motivation,* we uploaded a four-minute video that briefly described the concepts of reinforcement and punishment. The aim of the video was to **engage** students’ attention on the focal topic. To help students **explore** the topic in further, they were asked to respond to the following question: “After watching the video, can you think of other positive reinforcers, negative reinforcers, and punishment methods?” Students posted their opinions (**explained**) on a discussion forum. Students also used the WeChat app to ask questions of their peers or instructor.

**Fig. 6:**
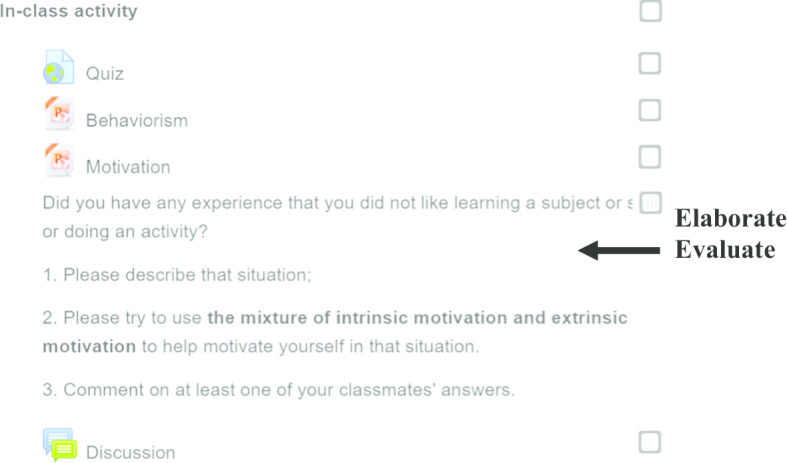
Example of an in-class activity in Course 2

During the subsequent face-to-face lesson (Fig. 6), the instructor facilitated whole class discussions using relevant questions to **elaborate** on the topics covered in the pre-class video. An example of a question used was ‘When should we employ positive reinforcement, negative reinforcement, or punishment?’ Based on the students’ responses, the instructor was able to provide more in-depth explanation of the subject matter, or correct any student misunderstanding. This will help enhance students’ comprehension of the subject content. The instructor also discussed the notion of intrinsic motivation (e.g., the self-determination theory). In addition to elaborating on the content, the instructor also **evaluated** the students’ understanding by asking students to complete small group discussion activities. An example of a small group discussion activity was ‘Did you have any experience where you did not like learning a subject or doing an activity? How would you motivate yourself in that situation? Please try to use a mixture of intrinsic and extrinsic motivation factors.’ Upon completion of the small group activity, students from each group presented their views to the whole class. The instructor, as well as the rest of the classmates provided feedback.

## Stage two: transforming conventional flipped classes into online flipped classes

The outbreak of COVID-19 inspired us to transform the two conventional flipped classes discussed above into fully online flipped classes. After careful consideration, the Zoom videoconferencing app was used for the synchronized online meetings (see Table 4). The whole transformation process took about one week with the bulk of the time was spent on exploring and testing the features of Zoom.

**Table 4:** Phases of the 5E framework and examples of activities in the online flipped classroom

5E phase	Component	Sample teaching and learning activities	Mode
Engage	Pre-class	Depict real-life situations (videos, case-studies); trigger learners’ interest in the subject matter	Online (asynchronous)
Explore	Pre-class	Learner completes self-reflection (forum) Learner asks questions if necessary (WeChat)	Online (asynchronous)
Explain	Pre-class	Learner articulates own opinions (forum) Learner participates in focused peer discussion on specific issues/topics (forum) Learner asks questions if necessary (WeChat)	Online (asynchronous)
Elaborate	In-class	Teacher-led class discussion to build understanding; teacher demonstration of strategies (if necessary) **(Zoom)**	**Online (synchronous)**
Evaluate	In-class	Student individual/group work and presentation, followed by teacher and peer feedback **(Zoom)**	**Online (synchronous)**

Zoom is a Web videoconferencing service that allows users to communicate online with individuals in real time via computer, tablet, or mobile device. We chose Zoom because of its ease of use (Kim 2017; Sutterlin 2018), its lower bandwidth requirements (Sutterlin 2018), and its ability to record and store sessions without recourse to third-party software (Archibald et al. 2019). More importantly, Zoom was chosen because its functions could easily support the implementation of our online flipped classroom. For instance, it allows instructors to easily create breakout rooms for group discussions. It also makes team-teaching possible by allowing more than one host and giving all of the hosts administrative capabilities such as sharing screens and remote control over shared screens (Johnston 2020).

To keep our online meetings secure, we activated the “*only authenticated users can join*” option. Specifically, we only allowed participants using our own university’s email domain to join the online meetings. In addition, we enabled the “*waiting room*” feature so that we could screen all of participants in the “*waiting room*” and admit only students officially enrolled in our classes into the online meeting. After all of the participants had entered, we then locked the meeting using the “*Lock the meeting*” feature. Once we had locked a meeting, no new participants could join.

The same learning materials used in the conventional flipped classes were used in the online flipped classes. Table 4 shows some of the teaching and learning activities. Students in the online flipped classes completed pre-class activities that were similar to those used in the conventional flipped classes, but these were not followed by face-to-face meetings, but by online meetings conducted on the Zoom videoconferencing app.

### Online flipped course 1: E-learning strategies

Like the conventional flipped course, the online flipped Course 1 consisted of eight sessions. The first seven sessions were flipped—students were encouraged to complete a set of pre-class sessions asynchronously (similar to Fig. 3). Students also used the WeChat MIM app to ask questions of their peers or instructors. However, unlike the conventional flipped approach, the “in-class” session for the online flipped students was conducted completely online through Zoom videoconferencing. In the final session (Session 8), the online flipped students also presented their work on Zoom. Each online “in-class” session lasted three hours—similar in duration to the in-class component of the conventional flipped format.

In the online synchronous “in-class” sessions, the instructor started by reminding students to switch on their webcams and to mute their microphones when not speaking. Next, the instructor lead a short class debriefing session to **elaborate** on the materials covered in the pre-class session. This was similar to the structure of the conventional flipped class format. For example, the instructor might discuss the students’ completed pre-class work and highlight the overall strengths and weaknesses. The main purpose of these short debriefing sessions was to clarify students’ initial doubts or misconceptions. Following the debriefing sessions, the instructor facilitated class discussions that delved deeper into the subject content. To **evaluate** students’ understanding of the materials, students were asked to work individually or participate in small group discussions on specific questions similar to those used in the conventional flipped classes. Students then presented their work online to the whole class, and received peer and instructor feedback.

**Fig. 7:**
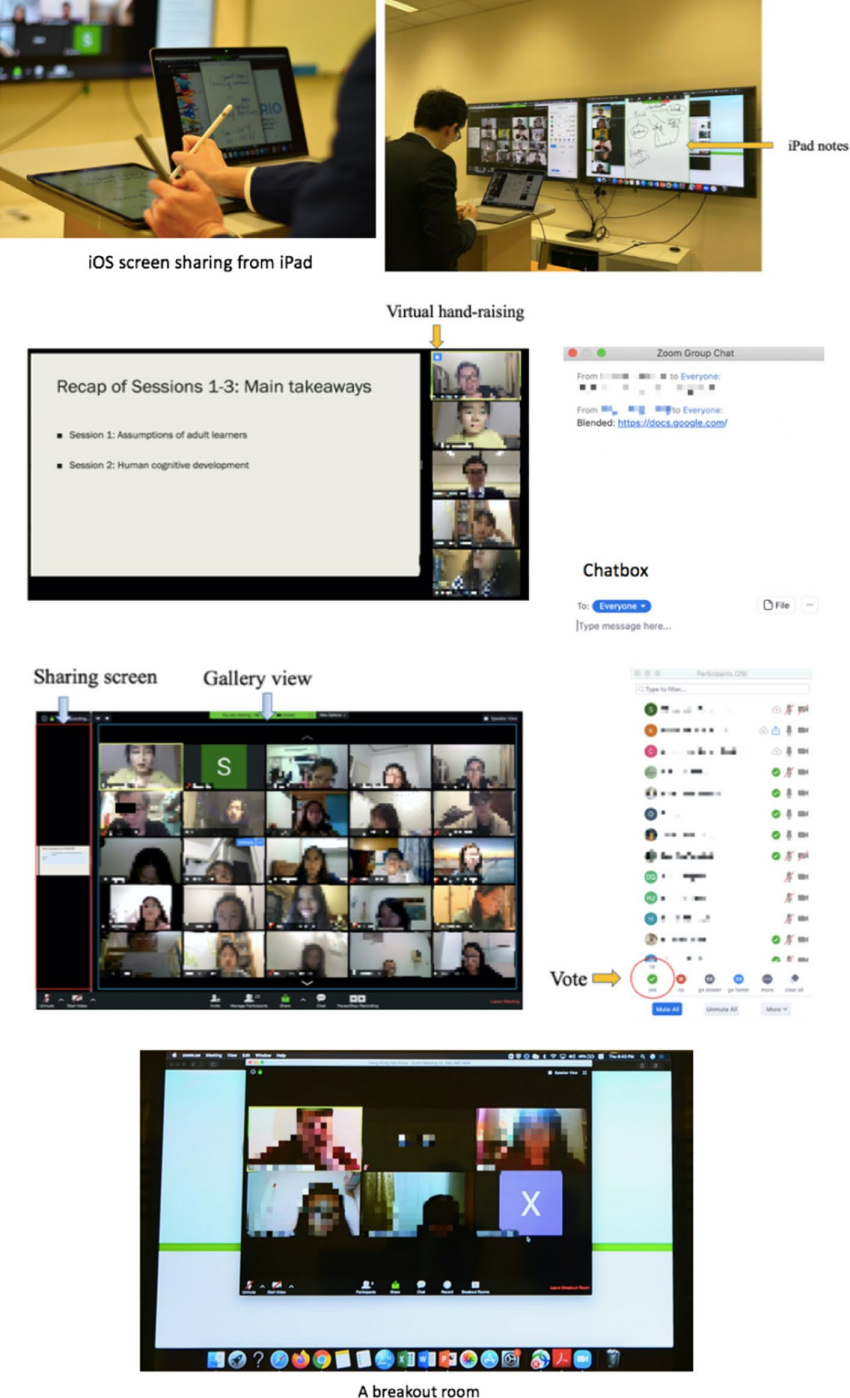
Examples of Zoom features used in Course 1

To engage the participants, the instructor used a number of features of the Zoom videoconferencing system. For example, the instructor posed questions during the whole class discussion and used the polling feature to rapidly collect and analyze student responses. The polling feature provided a function similar to a clicker or student response system. Based on the poll results, the instructor then addressed students’ misunderstandings. To enable small group discussions, the instructor used the breakout rooms feature of Zoom*.* Each student was assigned to one of several groups. Each group consisted of four to five students. Other students could not “drop” into other groups, but the instructor could drop into any group and participate in the discussions. When it was time for the small groups to return to the whole class, students would receive a time indicator reminding them that they were rejoining the whole class. Table 5 shows how the specific features of Zoom helped support the online “in-class” teaching and learning activities. Figure 7 illustrates some of the Zoom features used in the course.

**Table 5:** Examples of Zoom features supporting the online flipped classroom

Teaching and learning activities (example)	Zoom feature (*purpose*)
Teacher-led class discussion; teacher demonstration of strategies (if necessary)	Virtual hand-raising (*to allow individual students to ask questions*) Polling/voting (*to rapidly collect and analyze student responses to the questions*) Chat function (*to allow participants to ask questions or post comments*) iOS screen sharing from iPad (*to allow an instructor to write content on an iPad and share it with the participants*)
Group work and presentation, followed by teacher and peer feedback	Breakout rooms + broadcast features (*to assign participants to small groups for virtual group discussions* + *instructors used the broadcast feature to send relevant tips or suggestions to all of the small groups*) Screen-sharing (*to allow a participant to share his/her screen with other people*) Virtual hand-raising (*to allow individual students to ask questions*) Chat function (*to allow participants to ask questions or post comments*)

### Online flipped course 2: engaging adult learners

Similar to the conventional flipped course, the online flipped course had eight sessions. The pre-class and in-class activities used in the conventional flipped course were also used in the online flipped course (see Fig. 5 for an example of a pre-class activity). Students also used the WeChat MIM app to ask questions of their peers or instructors. The last three sessions were used for students’ online presentations via videoconferencing. Each online “in-class” session lasted three hours—similar in duration to the in-class component of the conventional flipped class. In the online synchronous “in-class” sessions, the instructor reminded students to switch on their webcams and to mute their microphones when not speaking. The instructor used the features of the Zoom videoconferencing system shown in Table 5 and Fig. 7.

## Results and discussion

Research question 1: What effect does the change from a conventional flipped classroom format to an online flipped format have on student learning performance?

### Conventional flipped versus online flipped course 1: E-learning strategies

To address Research Question 1, the learning outcomes of students in the conventional flipped Course 1 and the online flipped Course 1 were measured and compared. The main purpose of both courses was to teach students the skills needed to create an e-learning storyboard and to develop a fully online course based on the 5E framework on Moodle. At the beginning of both the conventional flipped and online flipped classes, students were surveyed if they had any experience creating storyboards or fully online courses. None of the students had any such prior experience. Therefore, we assumed that both groups of students had similar levels of prior knowledge/skill. Next, we used both groups of students’ final course marks as a measure of the student learning outcomes. The maximum final marks in the final assessment was 100.

**Fig. 8:**
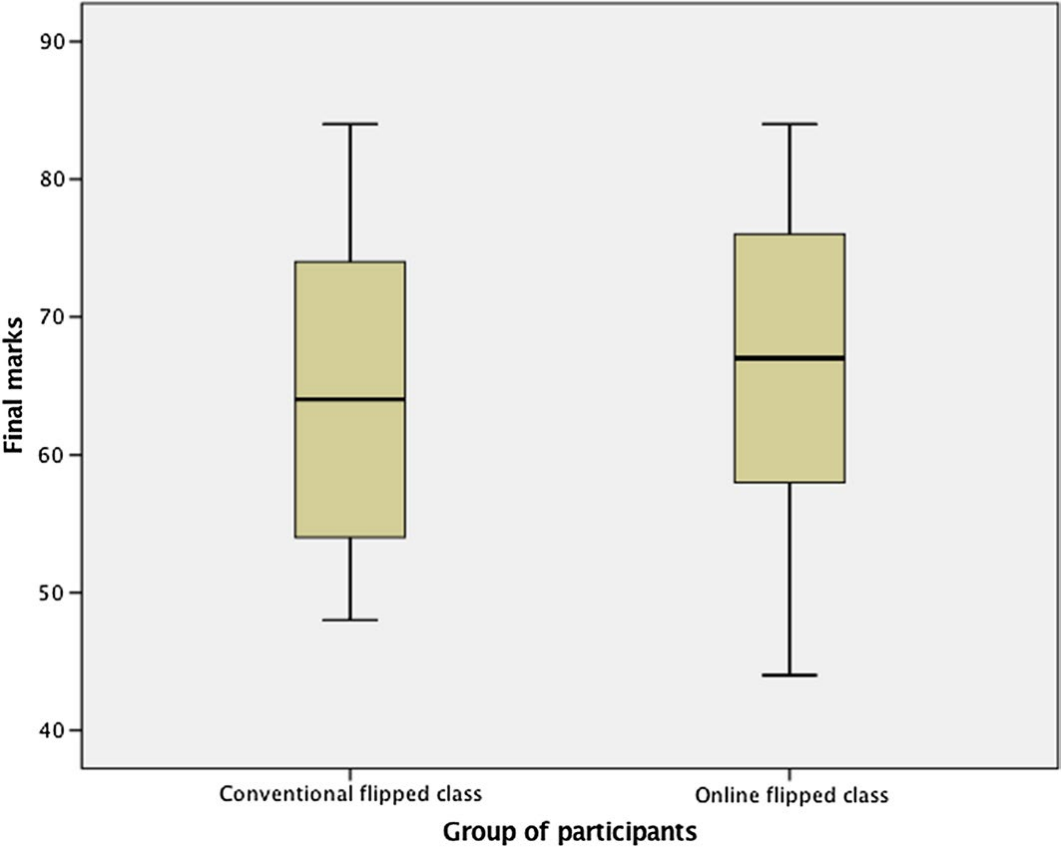
The boxplots of final marks in Course 1 for conventional flipped class and online flipped class

We first checked the normality of the final course marks data. If there were a significant deviation from normality, the Mann–Whitney U would be the most appropriate test for comparing the groups; otherwise, an independent samples *t*-test would be appropriate. The results showed that the course marks for both the conventional flipped (*W*(23) = 0.920, *p* = 0.068) and online flipped classes (*W*(26) = 0.964, *p* = 0.479) were normally distributed, as assessed by the Shapiro–Wilk’s test. There was also homogeneity in the variances for the course marks, as assessed by Levene’s test for equality of variances (*p* = 0.652). In addition, there were no outliers in the data, as assessed by an inspection of the boxplots (Fig. 8).

An independent-samples *t*-test was therefore conducted to determine if there were differences in the final marks of the conventional flipped and online flipped classes. The results suggested that online flipped participants (*M* = 66.00, SD = 11.63) performed as effectively as participants in the conventional flipped learning format (*M* = 65.04, SD = 11.80), *t*(47) = 0.285, *p* = 0.777.

### Conventional flipped versus online flipped course 2: engaging adult learners

The main purpose of both the conventional flipped and online flipped *Engaging Adult Learners* courses was to introduce students to the key characteristics of adult learners, the key principles of adult learning, and strategies for adult education. First, to test if there were any initial differences in students’ prior knowledge of the course content, a short quiz was administered to both groups at the start of the semester. The Mann–Whitney *U* test found no significant initial differences between the conventional flipped group (*Mdn* = 0) and the online flipped group (*Mdn* = 0.5), *U* = 218.5, *p* = 0.06.

**Fig. 9:**
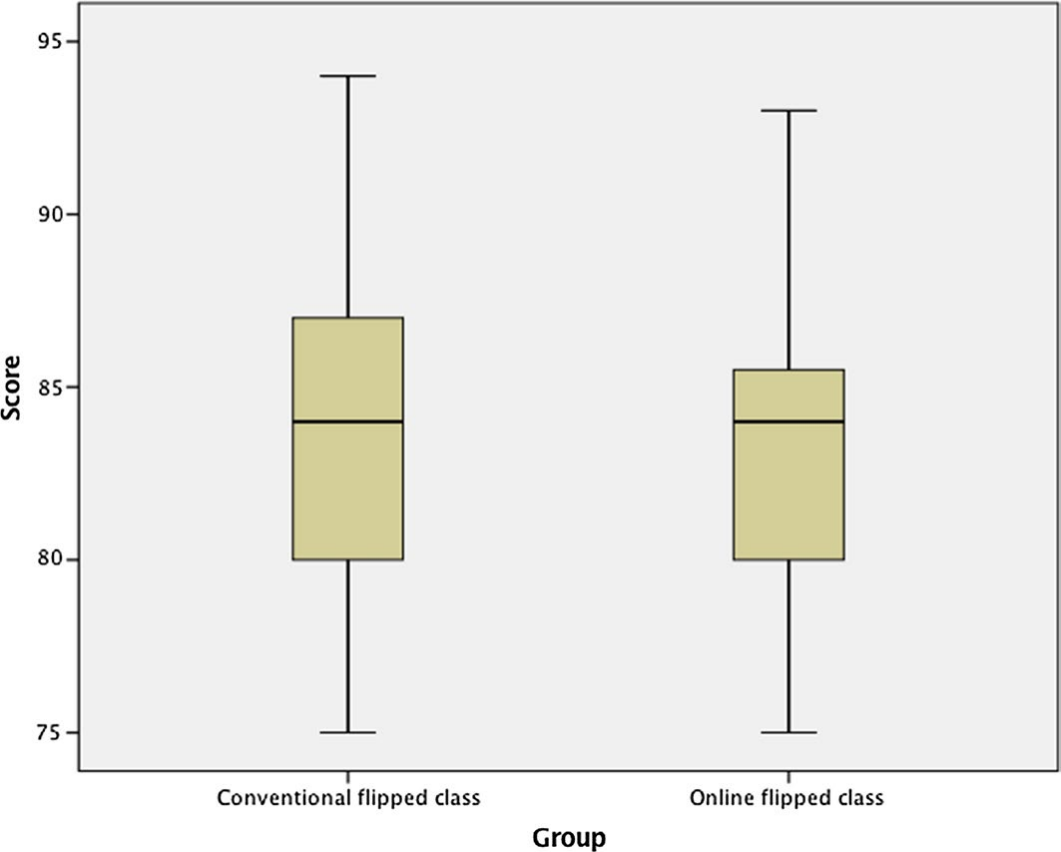
The boxplots of final marks in Course 2 for conventional flipped class and online flipped class

Next, we used the students’ final course marks as a measure of the student learning outcomes. The final assessment included individual written reflections on course topics and relevant articles, and a group demonstration of an adult-teaching strategy. The maximum final marks for the final assessment was 100. As in the above analysis, we first checked the normality of the final course mark data. The course marks for both the conventional flipped and online flipped classes were normally distributed, as assessed by Shapiro–Wilk’s test: *W*(25) = 0.963, *p* = 0.470 for the conventional flipped course and *W*(24) = 0.930, *p* = 0.096 for the online flipped course. There was also a homogeneity of variances, as assessed by Levene’s test for equality of variances (*p* = 0.304). In addition, there were no outliers in the data, as assessed by an inspection of the boxplots (Fig. 9).

We subsequently carried out an independent-samples t-test to examine if there was any significant difference in the final course marks of the conventional flipped and online flipped classes. The results suggested that online flipped learning participants (*M* = 83.25, SD = 4.56) performed as effectively as participants in the conventional flipped learning classes (*M* = 83.40, SD = 5.51), *t*(47) = 0.104, *p* = 0.918.

Research question 2: What are the good practices for videoconferencing-assisted online flipped classrooms, as perceived by students and/or teaching staff?

The analyses of the participants’ comments identified the following seven good practices for videoconferencing-assisted online flipped classrooms.

1. **Remind participants to mute their microphones when not speaking to eliminate undesirable background noise**. According to Gazzillo (2018), muting participants’ microphones allows the speaker to have center stage while eliminating the distraction of audio feedback. As one teaching staff member said, *.*
  It’s a good practice at the beginning to mute all of the participants by selecting the “Mute All” button at the bottom of the participants panel. This will eliminate all background noise (e.g., television sounds, audio feedback). I will then ask the participants to turn their audio back on if they wish to talk
  In terms of Zoom functionality, by pressing and holding the “space bar” allows the participants to temporarily switch on their microphone. We also ask the participants to install an AI-enabled application called “Krisp” to minimize the background noise of the participants.
2. **Remind participants before the online “in-class” session begins to switch on their webcams**. Webcams show a person’s face to other people on the video call, which can help to increase online social presence among classmates (Conrad and Donaldson 2011). Online social presence is positively correlated with student satisfaction and student perceived learning (Richardson et al. 2017). The participants also strongly prefer to see a face during instruction as it is perceived as more educational (Kizilcec et al. 2014). Students’ facial expressions are also a valuable source of feedback for the instructor to know whether the students could understand the subject matter (Sathik and Jonathan 2013). An instructor can use students’ facial expressions to determine whether to speed up, or slow down, or provide further elaborations. Feedback from the teaching staff included the following comments.
  It is important to ask students to turn on their cameras. Students will be more focused and interactive and teaching will be better when teachers can see students’ responses.
  As an instructor, I do not feel as if I’m talking to a wall when I can see some actual faces. Students also feel they are talking to someone rather than to an empty black screen. But it’s important to inform the students in advance to switch on their webcams so that they can do their hair properly or put on makeup beforehand—this was what some students actually told me!
  During teaching, seeing your students' faces will give you another form of feedback. For example, when they look confused or nod their heads, it allows me to fine-tune the delivery of the content. These reactions give me visual feedback on whether I need further explanations or examples to elaborate on the topic.
  Feedback from the students included the following comments.
  Showing our faces is really helpful as we can see our classmates’ faces and remember them. Also, it makes the class more alive because we can see their expressions. Showing our faces is very helpful! It can make me feel like I’m in a real class! I enjoy the feeling of having a class with my classmates.
  Turning on the camera helps us be more attentive in the online class.
  To avoid showing any undesirable background objects (e.g., a messy bedroom) during the video meeting, participants can choose to replace their actual background with a virtual background. The participants can easily do this using the Zoom virtual background feature.
3. **Manage the transition to the online flipped classroom approach for students**. Not every student will be familiar with the videoconferencing app or the flipped classroom approach. Therefore, to promote student buy-in of this new pedagogical approach, it is important for the staff to directly address two main issues: (a) the structure and activities of the online flipped course, and (b) the functions of the video conferencing app. Feedback from the students included the following comments.
  If teachers would like to use some functions in Zoom, they need to first help students get familiar with it. A brief introduction to Zoom at the beginning of the class is helpful.
  First, I informed the students that these two courses would have two components: a pre-class session and an online “in-class” session. This helped students understand the flipped approach better. Next, my teaching assistant and I conducted a short introduction to using Zoom online before the class began. This helped students get familiar with the features we would be using in Zoom.
  Constant fine-tuning is also a key element in managing the transition to the online flipped classroom. Asking the students what works and what doesn’t have become our practice every after the lesson. These comments allow us to rethink and re-plan for the next online synchronous session.
  Feedback from the teaching staff included the following comments.
  Having a technical-related orientation session before the actual class starts helps a lot for students who are not familiar with the videoconferencing tool.
4. **Instructors should use dual monitors to simulate, as close as possible, the look and feel of a face-to-face class—one monitor to view all the participants in “gallery view,” and the other to view the presentation material**. It is very useful for instructors and teaching assistants to use the dual-monitor display function, which allows the video layout and screen share content to be presented on two separate monitors. One monitor can be used to view the participants (up to 49) in “gallery view,” and the other to display the presentation materials. In the “gallery view,” the instructor can see thumbnail displays of all of the participants in a grid pattern that expands and contracts automatically as participants join and leave the meeting (Zoom Video Communications 2019). The use of a dual monitor feature is also useful for PowerPoint presentations and hiding notes from the participants. Feedback from the teaching staff included:
  During the preparation for this course, we would like to simulate, as close as possible, the look and feel of a face-to-face class. This thinking brought us to the dual monitor layout for our Zoom sessions. The first monitor is for the teaching assistant; in this case, it acts as a co-host for the Zoom session. The teaching assistant extends the computer screen to a monitor to show the participants’ faces or the “gallery view.” This monitor acts as a “classroom” in the traditional face-to-face class. During the session, this first monitor also serves as a tool for classroom management. This view is where the “chat” and “raise hand” functions can be seen. The second monitor is where the instructor places the presentation materials. This view acts as the projector in the traditional face-to-face class. Occasionally, we added a third screen, which is an iPad to do real-time annotation. This iPad can is a replacement of the conventional “whiteboard” in a face-to-face class.
5. **Activate and evaluate students’ pre-class learning with a short review.** At the beginning of the online “in-class” sessions, instructors should use short formative assessment methods (e.g., a quiz) to activate and evaluate students’ understanding of the pre-class activities. The activation of prior learning enhances student learning because it is the foundation for the new material presented in the classroom (Merrill 2002). Indeed, recent meta-analyses have suggested that flipped learning is more effective when formative assessments (e.g., quizzes or reviews) are used before and/or during class time (e.g., Hew and Lo 2018; Låg and Sæle 2019; Lo et al. 2017; van Alten et al. 2019). Students in this study reported positive benefits of using short formative assessments such as reviews or quizzes. Examples of student feedback include the following comments.
  I find the reviews at the beginning of the “in-class” sessions very helpful! It’s good to start from something we are familiar with, and then go to the new materials. The reviewing of pre-class work is great because we can know what points we do not understand well and how we can improve.
  The reviews helped me understand the issue more deeply. I could find out what my misunderstandings of the content are.
  I find the teachers’ explanation and review of the pre-class work helpful.
6. **Use an MIM app on mobile phones to foster quicker online response times and to communicate with students during their online breakout sessions**. Although students can ask questions via discussion forums or email, the asynchronicity of these apps creates a time lag between postings and replies which can discourage students from communicating with each other (Hew et al. 2018). In contrast, MIM apps such as WhatsApp and WeChat allow users to engage in quasi synchronous communications on their mobile phones. When communication needs are urgent, many students may only have their phones available. As soon as an MIM message is sent, a notification automatically shows up on the user’s phone screen, which encourages timely response (Hew et al. 2018; Rosenfeld et al. 2018). In addition, MIM is more popular than voice calls, emails, and even face-to-face communication among young people (Lenhart et al. 2010). As of March 2019, more than 41 million mobile instant messages are sent every minute (Clement 2019). Student feedback on using MIM in classrooms included the following comments.
  I like using MIM such as WeChat because it allows us to communicate with other people immediately.
  I enjoy using WeChat to ask questions and get immediate feedback from my classmates and teaching staff.
7. **Use a variety of presentation media as well as a variety of activities to sustain student interest**. No matter how interested a learner is in the topic of a presentation or discussion, that interest will wane in the face of monotony (Driscoll 2000). Therefore, it is recommended that instructors sustain student interest by varying the use of presentation media. Instructors, for example, can alternate the use of PowerPoint slides with digital handwriting on an iPad. The instructor in this study made the following comments.
  I find continual use of PowerPoint slides to be boring. It’s always the same style: a bullet list of information with some animations or pictures. I find it useful to sustain my students’ attention by writing on an iPad.
  Comments from the students were also positive.
  I find the instructor writing on an iPad helps to focus my attention better than PowerPoint slides.
  Writing on the iPad is like writing on a whiteboard in real face-to-face classrooms. It helps me develop a better understanding of the topic.
  Digital writing on an iPad can help learners see the progressive development of the subject content (Hulls 2005), and follow the instructor’s cognitive process better than pre-prepared PowerPoint presentations (Lee and Lim 2013). Writing on an iPad can also enable an instructor to immediately adjust his or her instruction in response to the students’ needs. Using digital writing can significantly improve students’ understanding of conceptual knowledge when compared to PowerPoint-based presentation lectures (Lee and Lim 2013).
  In addition to varying the presentation media, an instructor should also use different activities, including guest speakers, during the online class session. Feedback from the students included the following comments.
  The use of different functions in Zoom, such as breakout rooms for group activities, voting, and raising hands, is useful because they help us to be involved. It helps increase the learner-learner and learner-instructor interaction, which may be lacking in a fully online class.
  During the three-hour online class, we had not only the teacher’s explanations, but also had a guest speaker and online group discussions via breakout rooms, which made the class engaging.
  In this study, the instructor invited a United Kingdom-based practicing instructional designer as a guest speaker in the two online flipped courses to talk about her experience in developing e-learning courses and engaging adult learners. Guest speakers enhance students’ educational experience by giving them real-world knowledge (Metrejean and Zarzeski 2001). Guest speakers can offer students a different point of view, one that students may better understand. Guest speakers can also alleviate the monotony of listening to a single instructor.

## Conclusion

Amidst the burgeoning use of online learning during the unpredictable present, this study evaluates the efficacy of a videoconferencing*-*supported fully online flipped classroom. It compares student outcomes in four higher education classes: conventional flipped Course 1 versus online flipped Course 1, and conventional flipped Course 2 versus online flipped Course 2. Overall, this study makes three contributions to the literature on flipped classrooms. First, it provides a thick description of the development of the conventional flipped classroom approach based on the 5E framework, and the transformation of the conventional flipped classroom into a fully online flipped classroom. A thick description of the development of the flipped classrooms is provided to encourage replication by other researchers and practitioners. Second, our findings reveal that the online flipped classroom approach can be as effective as the conventional flipped classroom. Third, we identify seven good practices for using videoconferencing to support online flipped classrooms. This set of good practices can provide useful guidelines for other instructors who might be interested in implementing an online flipped approach.

One potential limitation of our study is that it was relatively short in duration (8 weeks). However, according to Fraenkel et al. (2014), some researchers do collect data within a fairly short time. A short-term data collection period enables researchers to collect and analyze data to see if an intervention is workable before committing to a longer study (Creswell 2015). We therefore urge future researchers to examine the use of videoconferencing*-*supported online flipped classrooms over a longer period of time, such as one year or more, to verify the results of this study.

Another interesting area for future work will be examining how instructors can support learners’ self-regulation during online flipped classroom (Cheng et al. 2019), as well as what strategies can best motivate students to complete the pre-class work.

## Data Availability

The anonymized datasets used and/or analysed during the current study are available from the corresponding author on reasonable request.
